# Sleep-promoting action of IIK7, a selective MT_2_ melatonin receptor agonist in the rat

**DOI:** 10.1016/j.neulet.2009.04.005

**Published:** 2009-06-26

**Authors:** Simon P. Fisher, David Sugden

**Affiliations:** Division of Reproduction and Endocrinology, School of Biomedical and Health Sciences, King's College London, London SE1 1UL, UK

**Keywords:** MT_2_, Sleep, Melatonin, IIK7, Melatonin agonist

## Abstract

Several novel melatonin receptor agonists, in addition to various formulations of melatonin itself, are either available or in development for the treatment of insomnia. Melatonin is thought to exert its effects principally through two high affinity, G-protein coupled receptors, MT_1_ and MT_2_, though it is not known which subtype is responsible for the sleep-promoting action. The present study used radiotelemetry to record EEG and EMG in un-restrained freely moving rats to monitor the sleep-wake behaviour and examined the acute sleep-promoting activity of an MT_2_ receptor subtype selective melatonin analog, IIK7. IIK7 is a full agonist at the MT_2_ receptor subtype but a partial agonist at the MT_1_ receptor and has ∼90-fold higher affinity for MT_2_ than MT_1_. Like melatonin, IIK7 (10 mg/kg i.p.) significantly reduced NREM sleep onset latency and transiently increased the time spent in NREM sleep, but did not alter REM sleep latency or the amount of REM sleep. An analysis of the EEG power spectrum showed no change in delta (1–4 Hz) or theta activity (5–8 Hz) following IIK7 administration. Core body temperature was slightly decreased (∼0.3 °C) by IIK7 compared to vehicle-treated rats. The acute and transient changes in the sleep-wake cycle mimic the changes seen with melatonin and suggest that its sleep-promoting activity is mediated by activation of the MT_2_ receptor subtype.

Melatonin synthesis in the pineal gland is controlled by the master circadian oscillator in the suprachiasmatic nucleus (SCN) which drives the release of noradrenaline from the sympathetic, autonomic neurones which innervate the gland. This multisynaptic neural pathway from the SCN to the pineal is activated each night in darkness resulting in an increase in melatonin synthesis which generates the dramatic diurnal rhythm in plasma melatonin seen in mammals [13]. The daily rhythm of melatonin plays an essential role in regulating the various changes in physiology which occur in photoperiodic (seasonal) mammals [14]. In mammals, melatonin may also play a role in organising circadian physiology; melatonin receptors are expressed in the SCN [7] and, with appropriate timing of administration, activation of these receptors can re-entrain the circadian clock both *in vivo* [19] and *in vitro* [20]. Indeed, melatonin administration can phase-shift and entrain human circadian rhythms [2,3], and the hormone has found increasing use as a treatment for sleep disorders. In fact, a prolonged release formulation of melatonin [Circadin^®^] and a melatonin agonist [Rozerem^®^] have been introduced recently for the treatment of insomnia [24,16], and other melatonin agonists or formulations are in development [11]. A recent clinical trial of another melatonin agonist (tasimelteon, VEC-162) reported beneficial effects on sleep latency and maintenance in healthy volunteers whose sleep was disrupted by a 5-h advance of the sleep-wake schedule [18]. Melatonin is likely effective because of both an acute sleep-promoting action and the ability to phase-shift circadian rhythms. Melatonin's actions are mediated by two G-protein coupled receptor subtypes, MT_1_ and MT_2_ receptors; evidence suggests that the phase-shifting response is mediated by the MT_2_ receptor subtype [6], but it is not known which receptor subtype is involved in the acute sleep-promoting action. The melatonin analogs tested for their effects on sleep so far have no selectivity for either subtype [9,18,23]. The present study investigated the acute effects of an MT_2_-subtype specific agonist [8] on sleep in a rat model previously shown to detect the acute sleep-promoting effects of melatonin and a clinically available melatonin agonist, ramelteon [9].

Adult male Sprague–Dawley rats (250–300 g, Harlan, UK) were housed under a 12 h:12 h light–dark cycle (lights on 07:00–19:00 h) at an ambient temperature of 21 ± 1 °C. Food and water were available *ab libitum*. All procedures were in accordance with the UK Animal Scientific Procedures Act (1986).

Rats were anaesthetized with ketamine (75 mg/kg i.p.; Fort Dodge Animal Health Ltd., Southampton, UK) combined with medetomidine (0.5 mg/kg i.p.; Pfizer, Sandwich, UK) and implanted with a radiotelemetry transmitter (Model TL11M2-F40-EET; Data Sciences International, St. Paul, USA) into the peritoneal cavity [9]. A pair of leads from the transmitter recorded cortical EEG via stainless steel screws placed on the skull (2 mm anterior to lambda on the right hand side, 2 mm anterior to bregma on the left hand side) with the screw tip resting on the dura. Two leads recorded EMG from the musculus cervicoauricularis. Core body temperature (*T*_c_) and locomotor activity data were obtained from the body of the radiotransmitter. Recovery from surgery was monitored by examination of the diurnal rhythm in *T*_c_; restoration of a robust diurnal rhythm in *T*_c_ (at least 2 weeks) was required before implanted rats were used in an experiment.

EEG, EMG, *T*_c_ and locomotor activity data were transmitted to a Data Sciences radio receiver (RPC-1) underneath each rat cage, then routed to a PC running DSI Dataquest Gold acquisition software. EEG and EMG data were continuously sampled at 500 Hz, with a 100 Hz filter cut-off, band-pass filtered (0.5–35 Hz for EEG and 80–120 Hz for EMG) and then used to identify vigilance states. Sleep/wake stages were scored as wake (W), NREM (non-rapid eye movement) sleep and REM (rapid eye movement) sleep for each 10 s epoch using a semi-automated approach as described previously [9]. Briefly, an initial automated step using SleepSign^®^ software (Kissei Comtec, Nagano, Japan) was followed by a review of all epochs by an experienced sleep scorer to eliminate errors in the assignment of W, NREM and REM stages using the EEG/EMG criteria defined previously [15]. NREM sleep onset latency was defined as the time from injection to the occurrence of 12 consecutive NREM epochs, and REM sleep onset latency as the time to the occurrence of 3 consecutive REM epochs. EEG power spectra for NREM and REM epochs were analysed offline using Fast Fourier Transformation (512 point, Hanning window, 0.5–20 Hz with 0.5 Hz resolution using SleepSign^®^). Rats were injected (i.p.) with the MT_2_ melatonin receptor agonist, IIK7 (10 mg/kg; dissolved in 2% DMSO, then diluted with 45%, w/v (2-hydroxypropyl)-beta-cyclodextrin) or vehicle (drug diluent). Treatments were administered in a fully balanced cross-over design with all rats receiving both drug and vehicle, with at least 1 week between treatments. Drug or vehicle was administered at 24:00 h (5 h after D onset). IIK7 (*N*-butanoyl-2-(2-methoxy-6*H*-isoindolo[2,1-a]indol-11-yl)ethanamine) was synthesized at University College London, UK [8].

IIK7 is one of a series of tetracyclic analogues of melatonin synthesized to explore the role of the indole nitrogen region of melatonin in the binding of the ligand to the melatonin receptor [8]. Information on these analogs, and other structure-activity data [25], indicate that the binding pocket of the MT_2_ receptor subtype is less stringent in the indole nitrogen/2-position of melatonin than the MT_1_ receptor subtype. As a result, compounds like IIK7 can be readily accommodated by the MT_2_ receptor but show weaker affinity for the MT_1_ receptor subtype. IIK7 has a 90-fold selectivity for MT_2_ (compared to MT_1_) in radioligand binding assays, with an MT_2_ receptor subtype affinity (p*K*_i_ 0.05 nM) about 6-fold higher than melatonin itself (0.33 nM) [22]. In NIH3T3 cells expressing the recombinant melatonin receptor subtypes, IIK7 is a full agonist at the MT_2_ receptor, but a less potent, partial agonist on the MT_1_ subtype (intrinsic activity ∼60% that of melatonin) [8]. In a well-established model of melatonin action, the *Xenopus laevis* melanophore aggregation assay [21], IIK7 is a full agonist with a potency (EC_50_ 4.2 ± 1.4 nM) 30-fold less than melatonin (EC_50_ 141 ± 20 pM) (Sugden and Bocianowska, unpublished data).

In the sleep studies, strict criteria were adopted to define NREM sleep onset latency (120 s of continuous NREM sleep) and a consistent routine was used for dosing in which rats were left undisturbed for 30–36 h before treatment. Before the start of the experiment all rats were fully accommodated to i.p. injection which was completed rapidly with minimal stress. This protocol gave NREM sleep onset latency values after vehicle injection which were very consistent, with a SEM of ∼10–15% of the mean with 6 rats per group.

Though IIK7 dissolved readily in DMSO it precipitated from solution when added to 0.9% (w/v) saline; higher percentages of DMSO did not improve solubility. Finally, a stable solution of IIK7 for injection was made by dissolving the compound in DMSO (final concentration 2%, v/v) followed by dilution in 45% (w/v) (2-hydroxypropyl)-beta-cyclodextrin in saline. Vehicle-treated rats received 2% (v/v) DMSO/45% (w/v) (2-hydroxypropyl)-beta-cyclodextrin in saline. NREM sleep onset and REM sleep onset latencies after injection of this vehicle (Fig. 1) were somewhat greater than after administration of the vehicle (1%, v/v DMSO/saline) used in earlier experiments [9]. These changes seem unlikely to be caused by the increased percentage of DMSO in the vehicle (2%, v/v vs. 1%, v/v) considering that DMSO up to 10% (v/v) in saline was reported to have no effect on sleep [5] and therefore may be related to the cyclodextrin vehicle. Nevertheless, even though the NREM sleep onset latency in this series of experimental rats was relatively high after vehicle treatment (72.5 ± 8.5 min), IIK7 significantly reduced NREM sleep onset time (44.8 ± 7.9 min, *p* = 0.027, *n* = 6; Fig. 1A). REM sleep onset latency was not altered by IIK7 (vehicle, 128.5 ± 16.8 min; IIK7, 132.1 ± 21.0 min, *n* = 6; Fig. 1C). The amount of time spent in NREM sleep after IIK7 was increased 250% during the first hour after dosing (vehicle, 3.9 ± 0.9 min; IIK7, 9.7 ± 2.3 min, *p* < 0.05, *n* = 6) reflecting the decrease in NREM onset time. No significant changes in NREM sleep were apparent during the second, third or fourth hours after IIK7 administration (Fig. 1B), and REM sleep time was not altered by IIK7 (Fig. 1D).

An analysis of the EEG power spectrum in all NREM and REM sleep epochs for the first 60 min (NREM) or 90 min (REM) after dosing with vehicle or IIK7 revealed no change in the power spectrum. Fig. 2A shows the mean power in delta (1–4 Hz) in IIK7- and vehicle-treated rats during NREM sleep (expressed as a percentage of the total power). Power in the delta band, also referred to as slow wave activity (SWA), is enhanced by sleep-promoting drugs such as zolpidem [1], and is also increased during the rebound NREM following sleep deprivation and is thought to represent a quantitative estimate of the homeostatic sleep process [10]. Previously, we have shown that neither melatonin nor ramelteon, administered to rats by i.p. injection at the same dose as IIK7, altered the NREM sleep EEG power spectrum [9]. IIK7, like melatonin and ramelteon [9], also failed to alter EEG power in the theta band (5–8 Hz) during REM sleep. IIK7 produced a small reduction in core body temperature (*T*_c_) after administration (Fig. 2C). During the 4 h period before treatment there was no difference in *T*_c_, between the two groups. After treatment, *T*_c_ of both groups increased transiently (ZT 17–18 h) but the rise in *T*_c_ after IIK7 injection was only ∼50% of that seen with vehicle. During the second hour of treatment (ZT 18–19 h) *T*_c_ fell in both groups but the fall in the group treated with IIK7 (Δ*T*_c_ −0.49 °C) was over twice as large as that measured in the vehicle-treated group (Δ*T*_c_ −0.24 °C). The fall in *T*_c_ slowed and then began to reverse in both treatment groups during the third and fourth hour of treatment. A fall in *T*_c_ is commonly seen after administration of hypnotic drugs.

This is the first study to investigate the melatonin receptor subtype involved in the acute sleep-promoting action of melatonin using a selective melatonin ligand. To date, IIK7 is one of only very few subtype selective melatonin receptor agonists which have been characterised *in vitro* using both radioligand binding assays to obtain receptor affinity data and cellular assays to determine functional activity on each receptor subtype. In radioligand binding experiments on recombinant cloned receptor subtypes, IIK7 has ∼90-fold greater affinity for the MT_2_ subtype than the MT_1_ subtype, and has slightly greater MT_2_ receptor subtype affinity (6-fold greater) than melatonin [8]. It also acts as a full agonist at the MT_2_ receptor when tested on cells (inhibition of forskolin-stimulated cyclic AMP in cells expressing recombinant MT_2_ receptors) with similar potency (3-fold greater) to melatonin, while at the MT_1_ receptor potency is 30-fold weaker and it has only partial agonist activity. This affinity and potency data led us to use IIK7 at a dose (10 mg/kg i.p.) which is the same as the dose of melatonin (and ramelteon) that we have shown previously significantly reduced NREM latency and increased NREM sleep duration in the first hour after administration [9]. Lower doses of melatonin (e.g. 3 mg/kg i.p.) or ramelteon (1 mg/kg i.p.) have no significant effect on NREM sleep onset latency or NREM duration after treatment [Fisher and Sugden, unpublished data]. Though the pharmacokinetics and metabolism of IIK7 are unknown, the acute effects of IIK7 were the primary focus of this study, and indeed IIK7, like melatonin, produced a significant decrease in NREM sleep onset latency with a significant, though brief, increase in NREM duration after administration. No change in REM onset latency, REM duration, delta activity during NREM sleep or theta power in the EEG during REM sleep were observed, which again is consistent with the effects seen after administration of melatonin or the non-subtype selective agonist, ramelteon [9]. This marked similarity in the profile of sleep changes suggests that IIK7 mimics the effect of melatonin administration on sleep. Our earlier work on the sleep-promoting effect of melatonin and ramelteon [9] did not measure *T*_c_, but others [12] have reported that administration of similar doses of melatonin during the night does produce a small fall in *T*_c_ similar to that seen with IIK7. The selectivity of IIK7 for the MT_2_ receptor suggests that this receptor is the subtype which mediates the acute sleep-promoting action of melatonin. A meeting abstract [17] reported that subcutaneous administration of another selective MT_2_ receptor agonist (UCM765) to rats reduced NREM latency and increased NREM sleep duration. However, UCM765 also delayed REM sleep onset as effectively as diazepam suggesting that this melatonin analog may act on additional receptors.

This study has shown that IIK7, an MT_2_ subtype selective agonist exhibits an acute sleep-promoting action in the rat which is very similar to that seen after administration of melatonin, suggesting that an MT_2_ melatonin receptor subtype mediates the acute hypnotic effect. The data highlight that the sleep-promoting effects observed after the administration of melatonin and related analogues are distinct from those produced by commonly used sedative-hypnotics. These include the non-benzodiazepine zolpidem, which, in contrast to melatoninergic compounds, alters the EEG power spectra of NREM sleep and can inhibit REM sleep [1]. Further studies will be necessary to confirm the role of the MT_2_ receptor subtype in this response including the use of MT_2_-subtype selective antagonists. Although a few antagonists with MT_2_-selectivity are currently available [4], the affinity of these compounds is lower than the affinity of melatonin itself which may pose practical difficulties in delivering sufficiently high doses of antagonists to effectively antagonise melatonin.

## Figures and Tables

**Fig. 1:**
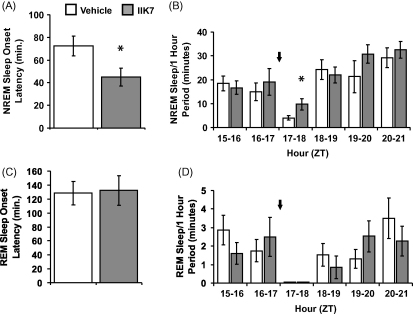
Effect of the MT_2_ selective agonist IIK7 on sleep in the rat. Rats housed under a 12 h:12 h L:D cycle (L on at 07:00 h) were injected (i.p.) with either vehicle or IIK7 (10 mg/kg) at 24:00 h in a balanced cross-over design. (A) NREM sleep onset latency. (B) NREM sleep duration in the 2 h before treatment, and the 4 h after injection. (C) REM sleep onset latency. (D) REM sleep duration in the 2 h before treatment, and the 4 h after injection. Arrows indicate time of IIK7 or vehicle administration. Each bar represents mean ± S.E.M. of data from 6 rats. *p < 0.05, significantly different from vehicle-treated rats.

**Fig. 2:**
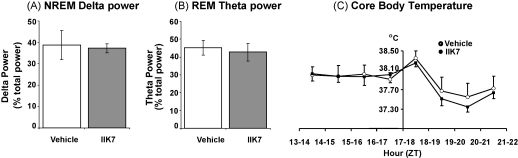
Effect of IIK7 administration on (A) delta power in the EEG during NREM sleep, (B) theta power during REM sleep, and (C) core body temperature (°C). All epochs scored as NREM (during the first 1 h) or REM (during the first 2 h) after injection of vehicle or IIK7 were analysed using the Fast Fourier Transform function of SleepSign^®^ from 0.5 to 20 Hz. For each rat, delta power (power in the delta range, 1–4 Hz) during NREM sleep and theta power (5–8 Hz) was calculated and expressed as a percentage of total EEG power in all of the NREM or REM epochs analysed, respectively. Mean delta (A) and theta (B) power ± S.E.M. data is shown. (C) Core body temperature was measured for 5 s every 5 min from 20:00 h to 04:00 h. Mean data for each hour of recording was averaged and the mean ± S.E.M. of these values for all 6 rats is shown.
